# Does swab type matter? Comparing methods for *Mannheimia haemolytica* recovery and upper respiratory microbiome characterization in feedlot cattle

**DOI:** 10.1186/s42523-022-00197-6

**Published:** 2022-08-13

**Authors:** William B. Crosby, Lee J. Pinnell, John T. Richeson, Cory Wolfe, Jake Castle, John Dustin Loy, Sheryl P. Gow, Keun Seok Seo, Sarah F. Capik, Amelia R. Woolums, Paul S. Morley

**Affiliations:** 1 Department of Pathobiology and Population Medicine, College of Veterinary Medicine, Mississippi State University, Mississippi State, MS USA; 2 Veterinary Education, Research, and Outreach Program, Texas A&M University, VERO Building, 3201 Russell Long Blvd., Canyon, TX 79015 USA; 3 Department of Agricultural Sciences, West Texas A&M University, Canyon, TX USA; 4 Nebraska Veterinary Diagnostic Center, School of Veterinary Medicine and Biomedical Sciences, University of Nebraska-Lincoln, Lincoln, NE USA; 5 Department of Large Animal Clinical Sciences, Western College of Veterinary Medicine, University of Saskatchewan, Saskatoon, SK Canada; 6 Department of Comparative Biological Sciences, College of Veterinary Medicine, Mississippi State University, Mississippi State, MS USA; 7 Texas A&M AgriLife Research, Amarillo, TX USA; 8 Department of Veterinary Pathobiology, Texas A&M University, College Station, TX USA

**Keywords:** Bovine respiratory disease, 16S rRNA gene sequencing, Antimicrobial resistance, Metagenomics, Culture, qPCR, Disease surveillance

## Abstract

**Background:**

Bovine respiratory disease (BRD) is caused by interactions among host, environment, and pathogens. One standard method for antemortem pathogen identification in cattle with BRD is deep-guarded nasopharyngeal swabbing, which is challenging, costly, and waste generating. The objective was to compare the ability to recover *Mannheimia haemolytica* and compare microbial community structure using 29.5 inch (74.9 cm) deep-guarded nasopharyngeal swabs, 16 inch (40.6 cm) unguarded proctology swabs, or 6 inch (15.2 cm) unguarded nasal swabs when characterized using culture, real time-qPCR, and 16S rRNA gene sequencing. Samples for aerobic culture, qPCR, and 16S rRNA gene sequencing were collected from the upper respiratory tract of cattle 2 weeks after feedlot arrival.

**Results:**

There was high concordance of culture and qPCR results for all swab types (results for 77% and 81% of sampled animals completely across all 3 swab types for culture and qPCR respectively). Microbial communities were highly similar among samples collected with different swab types, and differences identified relative to treatment for BRD were also similar. Positive qPCR results for *M. haemolytica* were highly concordant (81% agreed completely), but samples collected by deep-guarded swabbing had lower amounts of *Mh* DNA identified (Kruskal–Wallis analysis of variance on ranks, *P* < 0.05; Dunn-test for pairwise comparison with Benjamini–Hochberg correction, *P* < 0.05) and lower frequency of positive compared to nasal and proctology swabs (McNemar’s Chi-square test, *P* < 0.05).

**Conclusions:**

Though differences existed among different types of swabs collected from individual cattle, nasal swabs and proctology swabs offer comparable results to deep-guarded nasopharyngeal swabs when identifying and characterizing *M. haemolytica* by culture, 16S rRNA gene sequencing, and qPCR.

**Supplementary Information:**

The online version contains supplementary material available at 10.1186/s42523-022-00197-6.

## Background

Bovine respiratory disease (BRD) is one of the leading causes of morbidity and mortality in cattle, leading to significant economic loss in feedlot operations [1]. BRD is a complex disease involving the interaction between environmental factors, host immunity, and microbial pathogens [2, 3]. Though BRD is a multifactorial disease, the involvement of bacterial pathogens leads to antimicrobials being the primary treatment for suspected BRD, as well as being used for disease control and prevention [4, 5]. Widespread antimicrobial use to treat and control BRD has led to concerns over increased prevalence of antimicrobial resistance in isolates of BRD pathogens [6, 7].

Historically, the bacterial pathogen most commonly isolated through culture methods in North American feedlot cattle with BRD is *Mannheimia haemolytica* (*Mh*) [8], and it continues to be frequently associated with BRD [9]. Though *Mh* can be found in healthy animals [10], isolation of *Mh* from the upper respiratory tract in groups of animals affected by BRD is associated with isolation of *Mh* in the lungs [11]. Though aerobic culture is a mainstay of diagnostic procedures used to detect *Mh* which allows characterization through in vitro susceptibility testing and whole genome sequencing, use of culture-independent molecular techniques is beneficial to help decrease time for diagnosis, and to enhance detection of pathogens that are time consuming to confirm identity by culture [12]. Real-time qPCR has been reported for *Mh* [12], which allows for identification and quantification of *Mh* within a sample without the need for culture. Further, it is becoming increasingly important to study pathogens within the context of entire microbial communities, as is possible through 16S rRNA gene sequencing [13–15].

Perhaps, one of the the most common techniques for antemortem detection of BRD pathogens is the use of long (29.5-inch; 74.9 cm) double-guarded swabs [16–19] originally designed for uterine culture in mares. A perceived advantage of using these swabs is the ability to sample deep in the nasopharynx with less likelihood of contamination from the nares and rostral airways, given their guarded structure. Theoretically, use of these swabs allows more accurate localization of the anatomic source of important respiratory bacteria, as compared to use of short (6-inch) unguarded nasal swabs, which are commonly used to sample cattle for respiratory viral pathogens. In one study, nasopharyngeal culture showed higher agreement with lower airway sampling in calves affected by BRD than culture of the nasal passages [11]. However, other studies have shown high agreement between both nasal and nasopharyngeal culture of *Mh* when compared to lower airway culture in acutely ill dairy and beef calves [20, 21]. When evaluating the upper respiratory microbiome of healthy cattle, greater agreement has been shown between the nasopharyngeal bacterial community and the lung community than between the nasal passages and lungs [15]. However, deep nasopharyngeal sampling with the double-guarded swabs requires more technical skill and knowledge of anatomy than sampling with nasal swabs, and frequently requires firm restraint of the head. One possible alternative to these sampling techniques is a long (16-inch; 40.6 cm) large-tipped swab, designed for human proctological sampling. These swabs are long enough to reach the nasopharynx but more flexible than double-guarded swabs, potentially easing passage through the upper airways. The much larger swab head on the proctology swab (14 mm diameter × 35 mm length) also has the potential to collect a larger volume of respiratory secretions when compared to the smaller swab heads (5 mm diameter × 15 mm length) of the both long double-guarded swabs and short (6-inch; 15.2 cm) swabs commonly used for collection of microbial samples (Additioanl file 3: Figs. S1, S2). While either 16-inch proctology swabs ($363/500 count) [22] or 6-inch nasal swabs ($23/100 count) [23] are easier to use and less expensive, relative to the 29.5-inch double guarded swabs ($108/25 count) [24], the degree to which results for these proctology swabs agree with other sampling techniques has not been reported.

The objective of this study was to compare the use of long double guarded swabs of the nasopharynx, short swabs of the nasal passage, and long proctology swabs of the nasal passage and nasopharynx for the recovery and characterization of *Mh* in feedlot cattle when evaluated using culture for isolation and susceptibility testing, and culture-independent methods (qPCR, 16S rRNA gene sequencing). Importantly, this study addresses the abilities of these sample and testing strategies to characterize *Mh* in healthy animals and those with BRD.

## Methods

### Study population and sampling

Two groups each consisting of 60 beef-type steers and bulls were purchased from a livestock auction market located in central Texas, shipped to the West Texas A&M University Research Feedlot on May 14 and May 21, 2020, where they were enrolled in this study (n = 120). Upon arrival at the feedlot, cattle received an ear tag with an individual identification number and were processed following standard practices of many feedlots. Briefly, tildipirosin (Zuprevo, Intervet Inc., Summit, NJ), a long-acting macrolide, was administered to every animal at 4 mg/kg subcutaneously for BRD metaphylaxis. Animals were vaccinated against clostridial (Calvary 9, Merck Animal Health, Omaha, NE) and respiratory bacterial pathogens (Once PMH, Merck Animal Health, Omaha, NE), given a zeranol growth implant (Ralgro, Merck Animal Health, Summit, NJ) and given anthelminthic therapy with albendazole (Valbazen, Zoetis, Kalamazoo, MI) and ivermectin with corsulon (Ivermectin Plus, Durvet, Inc., Blue Springs, MO). Animals were also tested to identify any animals persistently infected with bovine viral diarrhea virus (BVD-PI) via antigen capture ELISA, and any BVD-PI animals were removed from the study. Bulls were castrated and given meloxicam at 1.1 mg/kg orally the day following metaphylaxis, vaccine, and anthelminthic administration (Additional file 4: Table S1).

Pens were monitored daily by trained feedlot personnel to identify animals with BRD, and animals were assigned a BRD clinical score of 0–4 based on visual signs of disease (Additional file 4: Table S2) [25]. Cattle were removed from pens if they had a clinical score of ≥ 2. Animals were classified as BRD positive if they had a rectal of temperature ≥ 40 °C and/or a clinical score of ≥ 3. Animals were treated for BRD with antimicrobials based on the feedlot protocol (Additional file 4: Table S2). The animals were on feed for 213 and 255 days for group 1 and group 2, respectively.

On day 14 after arrival, when a high prevalence of *M. haemolytica* shedding was expected [17], cattle were processed through a chute, where they were weighed and restrained for sampling. Six different nasal and nasopharyngeal samples (three from the left and three from the right) were obtained as previously described [11]. Briefly, the external nares were cleaned with a paper towel to remove superficial secretions and dirt, and both internal nasal passages were then swabbed with the 6-inch (15.2 cm) rayon fiber nasal swabs (NS) (SP130D, Starplex Scientific Corporation, St. Louis, MO); swabs were inserted approximately 2–3-inch into the nasal passages for sampling. After collecting nasal swabs, the 16-inch (40.6 cm) rayon fiber proctology swab (PS) (816-100, Puritan, Guilford, ME) or the 29.5 in (74.9 cm) cotton fiber deep-guarded swab (DG) (E9-5200, Continental Plastic, Delavan, WI) were used to sample the left and right nasal and nasopharyngeal passages by passing swabs to the caudal limit of the nasopharynx at the level of the palatopharyngeal arch; the order of collection of the proctology and deep-guarded swabs was randomized. All swabs collected via the left nostril were placed in modified Amies transport media (Starplex Scientific Corporation, St. Louis, MO) and used for aerobic bacterial culture, identification, and antimicrobial susceptibility testing. All swabs collected via the right nostril were placed in 100% ethanol to stabilize the microbial community structure and were used for DNA extraction and subsequent analyses with 16S rRNA gene sequencing and qPCR. All samples were kept on ice and transported to the laboratory for processing immediately after collection.

The unique animal ID was incorrectly recorded for two enrolled animals, which prevented extraction of corresponding data regarding animal weight and health records. Additionally, three swab samples intended for DNA analyses (two deep-guarded swab sample and one proctology swab sample), and one deep-guarded swab intended for culture were damaged during transport to the laboratory and could not be analyzed. DNA extraction from one deep-guarded swab sample failed, as well. These data are therefore missing from the results.

### Culture, microbial identification, and susceptibility testing

Swabs collected in modified Amies media were directly streaked onto one quadrant of a plate of tryptic soy agar (TSA) with 5% sheep blood (Remel, Lenexa, KS), and sterile disposable loops (Remel, Lenexa, KS) were used to streak the rest of the plate for bacterial isolation. Plates were incubated at 37 °C with 5% CO_2_. At 24 and 48 h of incubation, plates were monitored for growth consistent with *Mh* (2–3 mm, round, raised, light-grey, smooth, shiny colonies with faint β-hemolysis). If colonies consistent with such growth were present, catalase, oxidase, and indole tests were performed. If preliminary biochemical tests were consistent with *Mh* (catalase-positive, oxidase-positive, and indole-negative), a single colony was randomly selected by choosing the *Mh-*like colony closest to a mark made at a random position on the bottom of the media plate and subcultured onto a new blood agar plate and returned to the incubator at the above conditions. After 24 h, subcultures were monitored for colony phenotype and biochemical tests consistent with *Mh*. If present, 5–7 colonies were randomly selected with a sterile disposable loop and suspended into 1.5 mL of Brain Heart Infusion broth (B-D, Franklin Lakes, NJ) and 30% glycerol (Thermofisher, Waltham, MA). The same loop was then used to streak one half of another blood agar plate which was then incubated as described above for 24 h then shipped on ice to University of Nebraska-Lincoln Veterinary Diagnostic Center (UNL-VDC) to confirm identity and for antimicrobial susceptibility testing. Primary plates with no suspected *Mh* growth at 48 h were considered negative for *M. haemolytica.*

At UNL-VDC, a single colony from the shipped plate was subcultured overnight on blood agar to ensure pure growth which was then used to confirm *Mh* identification and antimicrobial susceptibility testing. Matrix assisted laser desorption-ionization time-of-flight mass spectroscopy (MALDI-TOF) was used to confirm *Mh* identity as well as MALDI-TOF biomarker-based genotyping of *Mh* isolates [26].

Antimicrobial susceptibility testing was performed at UNL-VDC using semi-automated broth microdilution via the Sensititre system (ThermoFisher, Waltham, MA) and the bovine/porcine panel containing gamithromycin and tildipirosin (BOPO7F Vet AST Plate, ThermoFisher, Waltham, MA). Results were interpreted according to breakpoints for *Mh* in BRD from the Clinical and Laboratory Standards Institute [27]. Isolates were characterized as multidrug resistant (MDR) if they were not susceptible to antimicrobial(s) from ≥ 3 antimicrobial classes [28]. Because the concentration range for ampicillin on the BOPO7 plate does not include CLSI breakpoints, only minimum inhibitory concentration (MIC) was recorded, and ampicillin resistance classification was not included in determination of isolates as MDR.

### DNA extraction

DNA was isolated from swab samples using a QIAamp PowerFecal DNA Kit (Qiagen, Hilden, Germany) according to the manufacturer’s instructions. Following isolation, DNA was quantified (ng/uL) using a Qubit Flex fluorometer (ThermoFisher, Waltham, MA).

### qPCR sample preparation and reaction conditions

From the extracted DNA, two 400 ng DNA aliquots were sent to Mississippi State University for qPCR. Samples from one aliquot were diluted in Low-Tris TE buffer to an estimated final concentration of 8 ng/μL. Final concentrations were measured on a Qubit 4 fluorometer (Thermofisher, Waltham, MA), and the mean concentration sample DNA templates was 6.73 ± 2.00 ng/μL. A standard curve for DNA quantification was made using 8, tenfold dilutions (maximum = 1.8 ng/μL, minimum = 1.8 × 10^−7^ ng/μL) of DNA extracted from a pure culure of *Mh* confirmed by Sensititre GNID (Thermofisher, Waltham, MA). Nine replicates of each standard were made and run using a QuantStudio 3 Real-Time PCR instrument (Thermofisher, Waltham, MA) with the following reaction mixture: 7 μL of *Mh* DNA standard, 10 μL of PerfeCTa SYBR Green FastMix Low ROX (Quantabio, Beverly, MA), 1 μL each of forward (F) and reverse (R) primer for *Mh* leukotoxin D gene *(lktD)* (F-CTGCAACAAAGCCGATATCTTT, R-TACGACTGCTGAAACCTTGAT) [12], and molecular grade H_2_O to reach a final volume of 20 μL. Amplification occurred under the following conditions: 95 °C for 5 min, then 45 cycles of 95 °C for 15 s, and 60 °C for 45 s. QuantStudio Design and Analysis Software v. 1.5.1 default settings (Thermofisher, Waltham, MA) were used to determine cycles of quantification (C_q_) threshold of replicates, melt-curve analysis, and other quality control checks, and results were exported to a spreadsheet for analysis using Excel for Mac Version 16.5 (Microsoft). A standard curve of C_q_ v. log_10_(ng of DNA) was created for calculation of the mass of *Mh* DNA. The lowest mass of DNA with SD C_q_ ≤ 0.5 was considered the limit of detection.

For sample plates, all reactions were run in triplicate using a QuantStudio 3 Real-Time PCR instrument (Thermofisher, Waltham, MA) and the following reaction mixture: 40 ng (mean = 41.5 ng, SD = 4.7 ng) of sample DNA, 10 μL of PerfeCTa SYBR Green FastMix Low ROX (Quantabio, Beverly, MA), 1 μL each of F and R primer for *Mh lktD* gene [12], and molecular grade H_2_O to reach a final volume of 20 μL. A smaller calibration curve using five, tenfold dilutions of DNA extracted from pure growth of *Mh* confirmed by Sensititre GNID (Thermofisher, Waltham, MA) were included on each 96-well MicroAmp plate (4316,813, Thermofisher, Waltham, MA) to confirm reaction efficiency between 90–110%. Any plates with reaction efficiency less than 90% or greater than 110% were rerun. Also included on each plate were negative controls consisting of reaction mixture of molecular grade H_2_O in place of template DNA. Additionally, controls with no primer added, and no master mix controls added were included. Amplification occurred under the same conditions as described above.

C_q_ was determined using QuantStudio Experiment Design and Analysis Software v. 1.5.1, then reviewed manually. Melt curves were used to check reaction specificity. Samples with undetermined C_q_, with C_q_ SD greater than 0.5, with melt curves indicating non-specific binding, and/or with calculated DNA mass of less than limit of detection determined from overall standard curve, were considered to have no amplification. Mass of *Mh* DNA was calculated from standard curve and was logarithmically transformed for statistical analysis of geometric means. For samples with no amplification, the mass of *Mh* DNA was recorded as 1 × 10^4^ ng, a non-zero number below the limit of quantification. Log_10_ (*Mh* DNA) per ng of DNA in reaction was recorded and used as outcome variable for statistical analysis.

### 16S rRNA library preparation, and sequencing

Preparation of libraries for sequencing of the V3-V4 region of 16S rRNA was conducted as previously described (Illumina, 2013). The V3-V4 region of the 16S rRNA gene was amplified using the 341F (5′-CCTACGGGNGGCWGCAG-3′) and 805R (5′-GACTACHVGGGTATCTAATCC-3′) primer pair (Integrated DNA Technologies, Inc, Coralville, IA) and sequencing libraries were prepared using the Nextera IDT kit (Illumina, San Diego, CA) [29]. The resulting pooled amplicon library was sequenced on an Illumina NovaSeq instrument using paired-end chemistry (2 × 250 bp) at the University of Colorado Anschutz Medical Campus’ Genomics and Microarray Core.

### Bioinformatics and statistics

Demultiplexed paired-end reads generated from 16S rRNA gene sequencing were imported in QIIME2 version 2020.11 [30]. Amplicon sequence variants (ASVs) were generated using DADA2 [31], which was also used to filter reads for quality, remove chimeric sequences, and merge overlapping paired-end reads. Forward and reverse reads were truncated at 248 bp and 250 bp, respectively. Taxonomy was assigned using a Naïve Bayes classifier trained on the Greengenes version 13_8 99% OTUs database [32], where sequences had been trimmed to include only the base pairs from the V3–V4 region bound by the 341F/805R primer pair. Reads mapping to chloroplast and mitochondrial sequences were filtered from the representative sequences and ASV table using the ‘filter-seqs’ and ‘filter-table’ functions, and a midpoint-rooted phylogenetic tree was generated using the ‘q2-phylogeny’ pipeline with default settings, which was used to calculate phylogeny-based diversity metrics. Data and metadata were then imported into phyloseq [33] using the ‘import_biom’ and ‘import_qiime_sample_data’ functions and merged into a phyloseq object. The proportion of reads mapped to each taxonomic rank can be found in Additional file 4: Table S3. ASV counts for each sample were then normalized using cumulative sum scaling [34] and beta-diversity was analyzed using a generalized UniFrac distance matrix [35, 36]. From these distances, principal coordinates analysis (PCoA) was performed and plotted, and a permutational multivariate analysis of variance (PERMANOVA) was used to test for significant differences in community structure using the vegan [37] and pairwiseAdonis [38] packages. To ensure significant differences were not the result of unequal dispersion of variability between groups, permutational analyses of dispersion (PERMDISP) were conducted for all significant PERMANOVA outcomes using the vegan package. Further, the relative abundances of ASVs within each sample were calculated and plotted using phyloseq. Differences in relative abundance were tested using a pairwise Wilcoxon rank-sum test with a Benjamini–Hochberg correction for multiple comparisons in R version 3.6.0.

Summary statistics of arrival weight, number of animals treated for BRD overall and number treated at time of sampling, and days on feed (DOF) until their first BRD treatment were calculated using R version 4.0.3 [39]. Comparisons between the two sampling groups were made using Wilcoxon rank-sum test for continuous outcome variables (arrival weight and DOF until first treatment) and Chi-square test for binary response variables (treatment for BRD during feeding period and treatment for BRD at the time of sampling) using the rstatix and stats packages in R [39, 40]. Cochran’s Q test was used to compare isolation of *Mh* by swab type using SAS software v 9.4 (SAS Institute, Cary, NC). If differences were found using Cochran’s Q test, pairwise comparisons using McNemar’s Chi-square test were performed with the rstatix package [40].

Comparisons of log_10_(ng *Mh* DNA) per nanogram of DNA among swab types and *Mh* culture status were assessed using Kruskal–Wallis analysis of variance by ranks using rstatix, stats, and diplyr packages [39–41]. If differences were found, pairwise comparisons were tested with a Dunn test with Benjamini–Hochberg correction for multiple comparisons. Differences in qPCR amplification (Yes or No) rates among swab types were tested using Cochran’s Q test in SAS software v 9.4 (SAS Institute, Cary, NC), with post hoc comparisons tested with pairwise McNemar’s Chi-square in rstatix.

## Results

### Cattle population

At the time of sampling (14 DOF), the mean body weight of all animals was 261.2 kg (SD = 12.2 kg). A total of 36% (43/118) of calves were treated for BRD at least once during the feeding period (Additional file 2). There was a greater number of calves treated for BRD in the first group than the second group of calves, with 50.0% (30/60) and 22.4% (13/58) treated respectively (Table 1; Chi-square test, *P* = 0.003). The median day until first treatment for all sampled calves was 10 days, and there was not a statistically detectable difference between groups (Wilcoxon rank-sum test, *P* = 0.188). Only one calf received his first antimicrobial treatment for BRD after day 40 (Additional file 3: Figs. S3–S5).

**Table 1:** Descriptive statistics of weight, number of animals treated, and days to first treatment

Group	Animals (n)	Mean weight (kg)	SD weight (kg)	Animals treated total (n)	Median days to first treatment	Range of days to first treatment
Group 1	60	260.1	11.7	30^a^	9.5	6–116
Group 2	58*	262.3	12.8	13^b^	13	6–22
All	118*	261.2	12.2	43	10	6–116

Values in the same column width different superscripts indicate significant difference (Chi-square; ab, *P* = 0.003)

*Unique animal IDs were mis-recorded for two cattle preventing the ability to link feedlot records regarding weight and BRD occurrence

### Culture results and isolate characterization

**Fig. 1:**
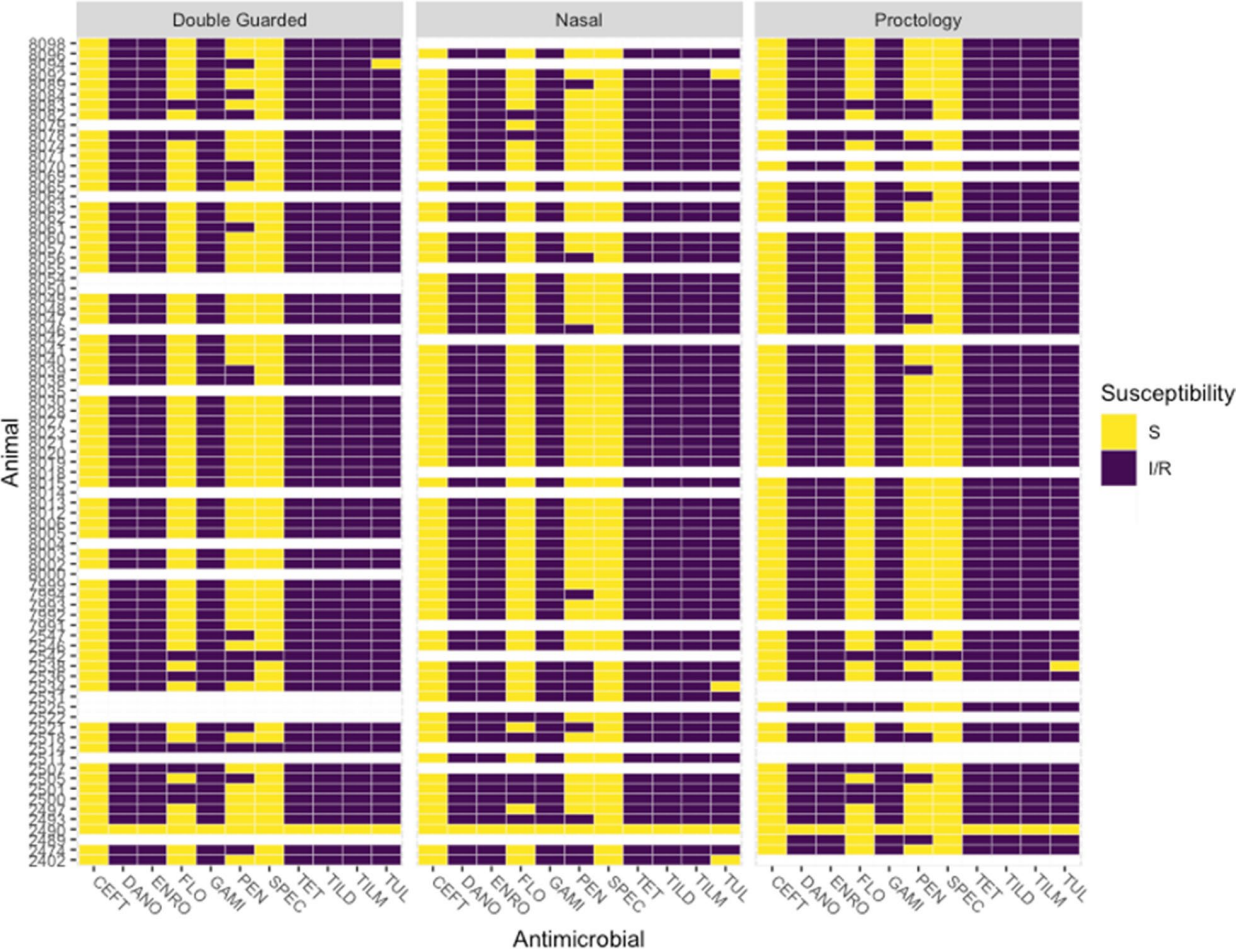
Antimicrobial resistance patterns of *M. haemolytica* isolated from each calf separated by swab type. Each isolate is identified by the calf from which it was isolated. Blank lines indicate calves that did not have *M. haemolytica* isolated from that swab, though it was *M. haemolytica* positive via (an)other swab type(s). *S* susceptible, *I/R* intermediate or resistant, *CEFT* ceftiofur, *DANO* danofloxacin, *ENRO* enrofloxacin, *GAMI* gamithromycin, *PEN* penicillin, *SPEC* spectinomycin, *TET* tetracycline, *TILD* tildipirosin, *TILM* tilmicosin, *TUL* tulathromycin

Overall, *Mh* was isolated by culture from 67.5% (81/120) of cattle: 55.0% (67/119) of DG, 56.3% (66/120) of NS, and 56.7% (68/120) of PS, with significantly higher frequency of *Mh* isolation in group 1 than group 2 for each swab type (Additional file 4: Table S4; Chi-square test, *P* < 0.05). All 201 *Mh* isolates were identified as genotype 2, and nearly all isolates were MDR (98.5%, 198/201; Additional file 2, Fig. 1). Three isolates were pansusceptible, and these isolates were from the different swabs from the same animal (Animal 2490, Fig. 1). AMR was similar for *Mh* collected from the same animals (Fig. 1), though there were slight differences in penicillin resistance among swabs isolated from the same animal. Frequency of *Mh* isolation was not statistically different among swab types (Additional file 4: Table S3; Cochran’s Q test, *P* = 0.86). There was complete concordance in culture results for the 3 sampling methods for 77% of cattle (92/119); two concordant positive and 1 discordant negative result was found in 11% of cattle (13/119), and 1 discordant positive result was identified in the remaining 12% of cattle (14/119) (Table 2).

**Table 2:** Concordance of swab types for culture and qPCR with culture and qPCR pattern of swabs

Concordance	Isolation pattern (DG, NS, PS)	Culture frequency	Culture percentage (%)	qPCR frequency	qPCR percentage (%)
Full	YYY	53	77.3	54	81.9
	NNN	39		41	
Two yes	YYN	3	10.9	2	12.1
	YNY	5		1	
	NYY	5		11	
Two no	NNY	4	11.8	1	6.0
	NYN	4		5	
	YNN	6		1	
	Total	119*	100	116*	99.9

Percentage is out of total swabs with results from all 3 swab types

*Samples from 1 swab for culture (DG) and 4 swabs for qPCR (3 DG and 1 PS) were damaged in transport or failed DNA extraction so samples from these animals were not used in concordance analysis. *DG* double-guarded swab, *NS* nasal swab, *PS* proctology swab, *Y* culture/qPCR positive, *N* culture/qPCR negative

### qPCR

#### Standard curve

C_q_ and SD of standards is included in Additional file 1. The lowest concentration standard had 6 out of 9 replicates that did not amplify and the second lowest concentration had high C_q_ SD (0.770), so the limit of detection was considered to be 1.26 × 10^−4^ ng. The slope and y-intercept of the C_q_ versus log(*Mh* DNA) was − 3.3998 and 17.3361, respectively. The efficiency was 96.8% and R^2^ was 0.9981 (Additional file 1).

#### qPCR of samples

**Fig. 2:**
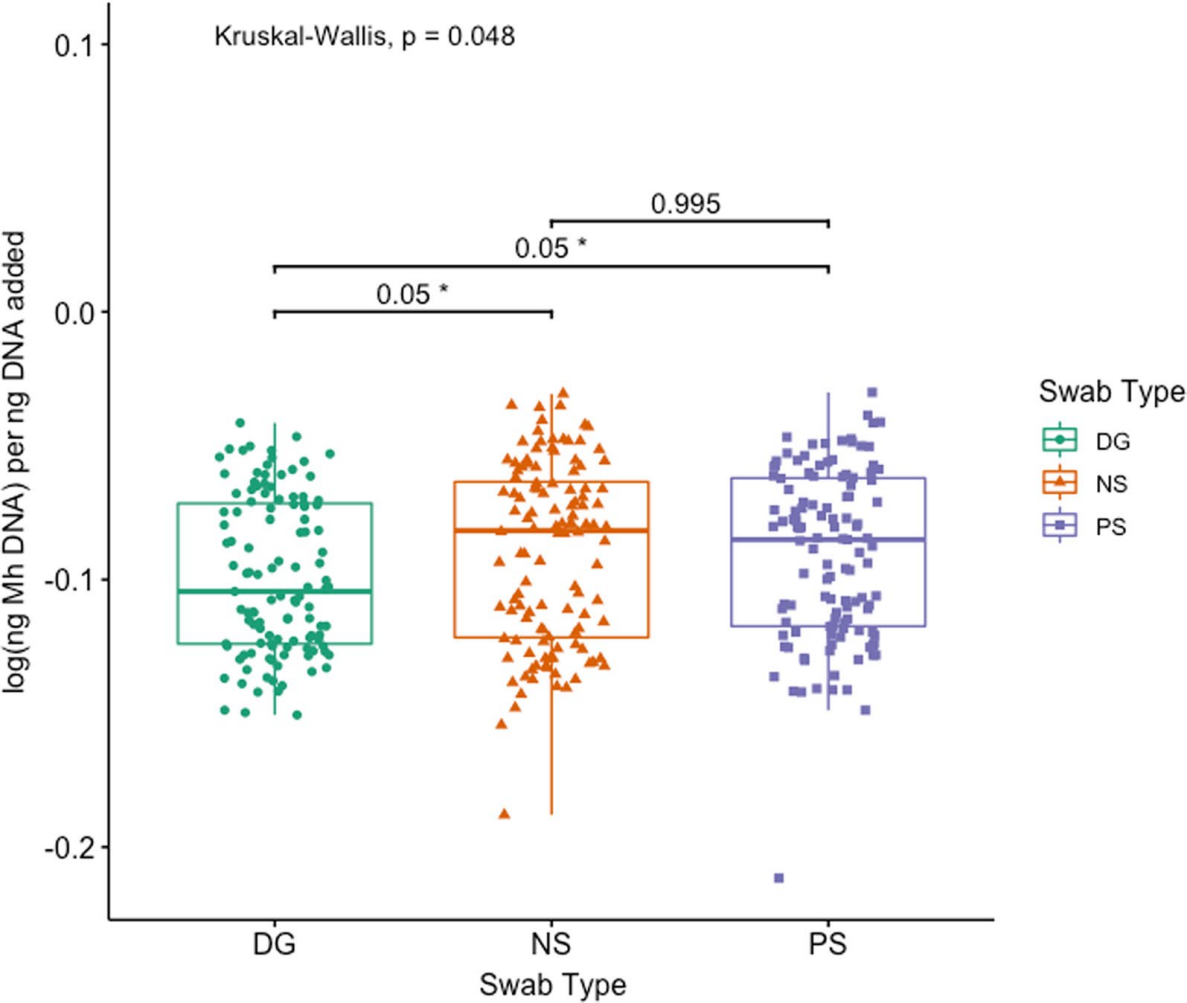
Box and whisker of log(ng *Mh* DNA) per ng DNA added vs. swab type. (Kruskal–Wallis test, *P* < 0.05; *pairwise Dunn test with Benjamini–Hochberg correction, *P* < 0.05). Key: DG = double guarded swab, NS = nasal swab, and PS = proctology swab

**Fig. 3:**
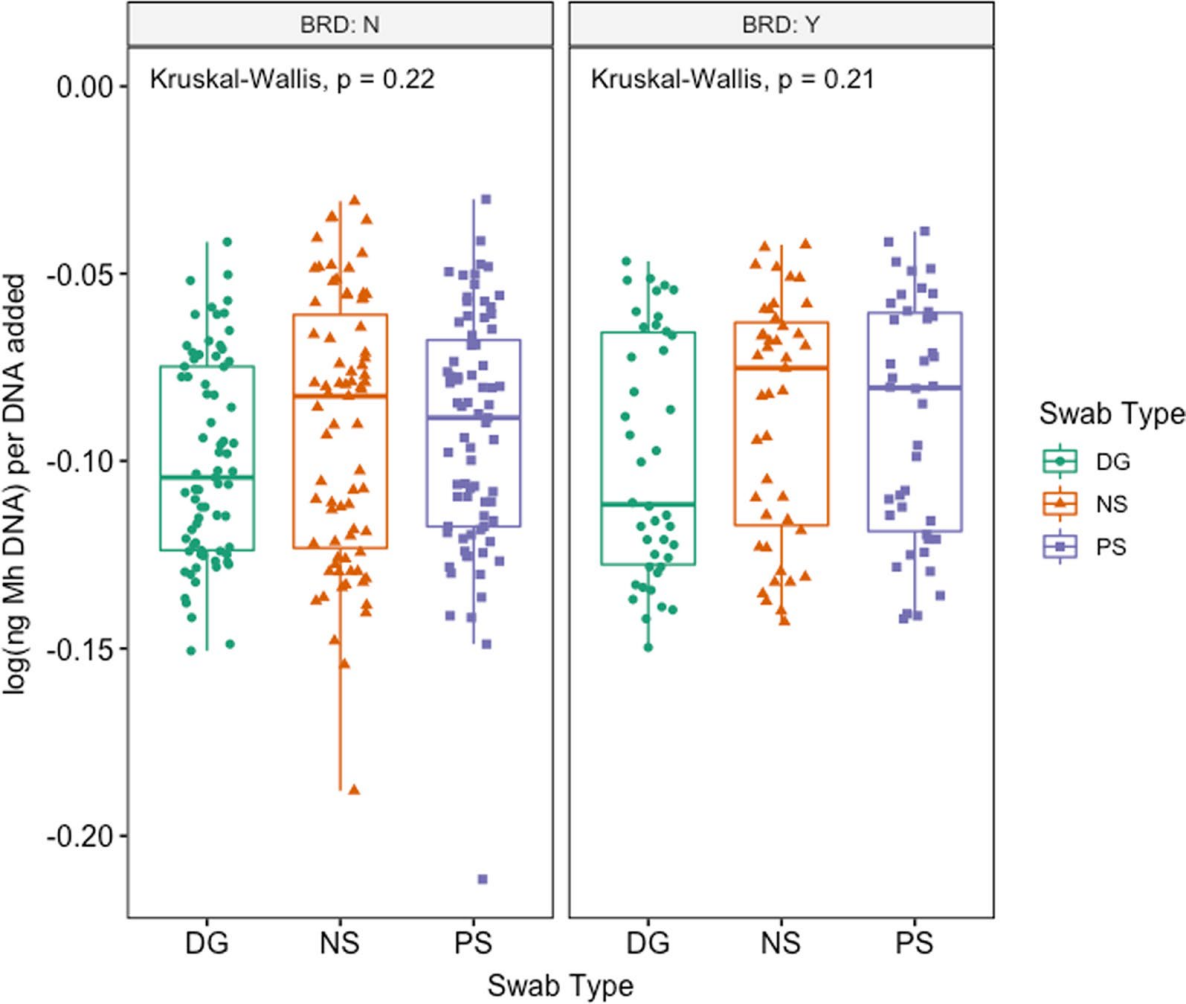
Box and whisker of log(ng *Mh* DNA) per ng DNA added vs. swab type, separated by BRD treatment (Kruskal–Wallis test, *P* > 0.05). Key: DG = double guarded swab, NS = nasal swab, and PS = proctology swab, BRD:Y = BRD positive, BRD:N = BRD negative

**Fig. 4:**
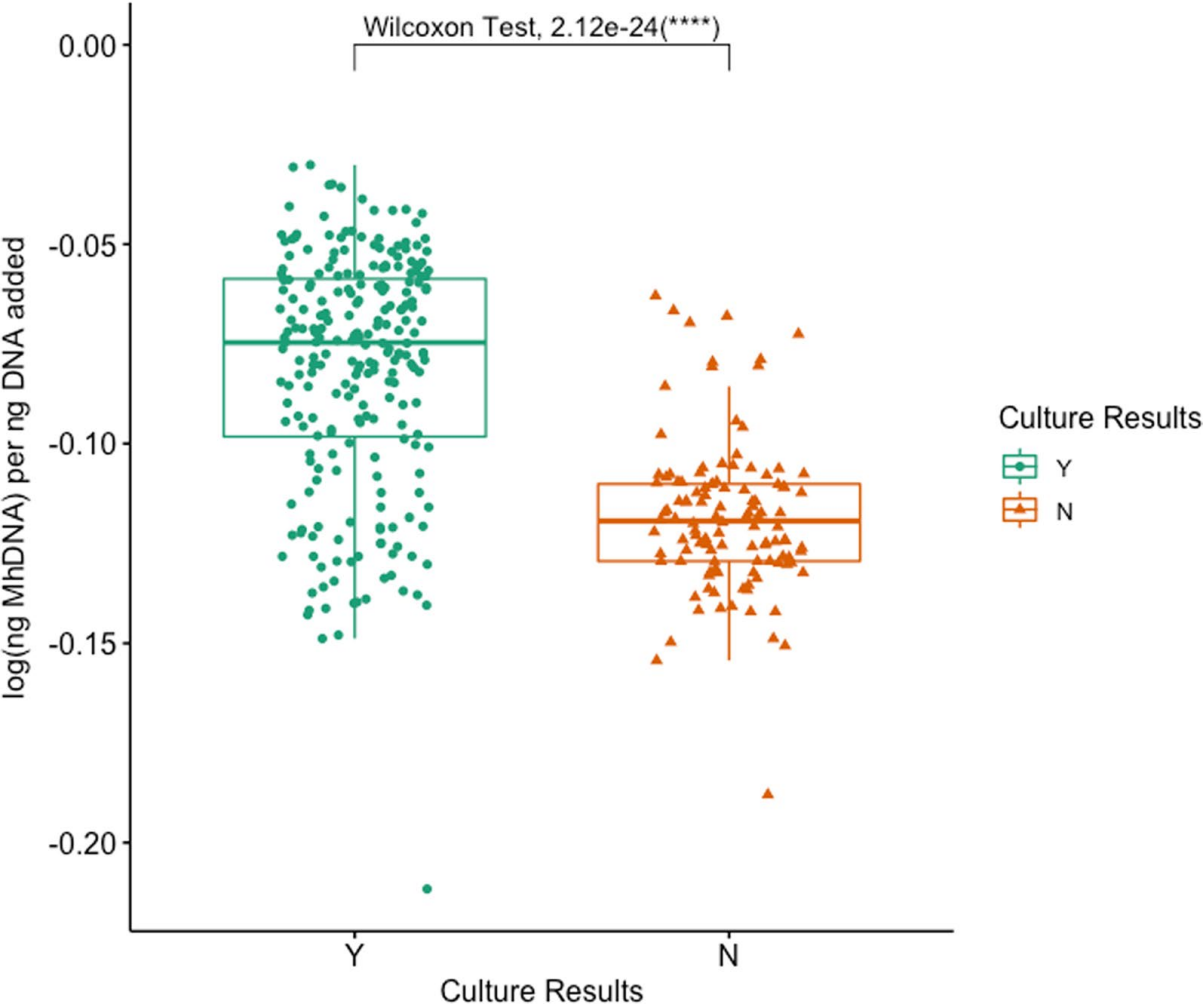
Box and whisker of log(ng *Mh* DNA) per ng DNA added vs. culture results (Wilcoxon rank-sum test, *P* < 0.05)

**Fig. 5:**
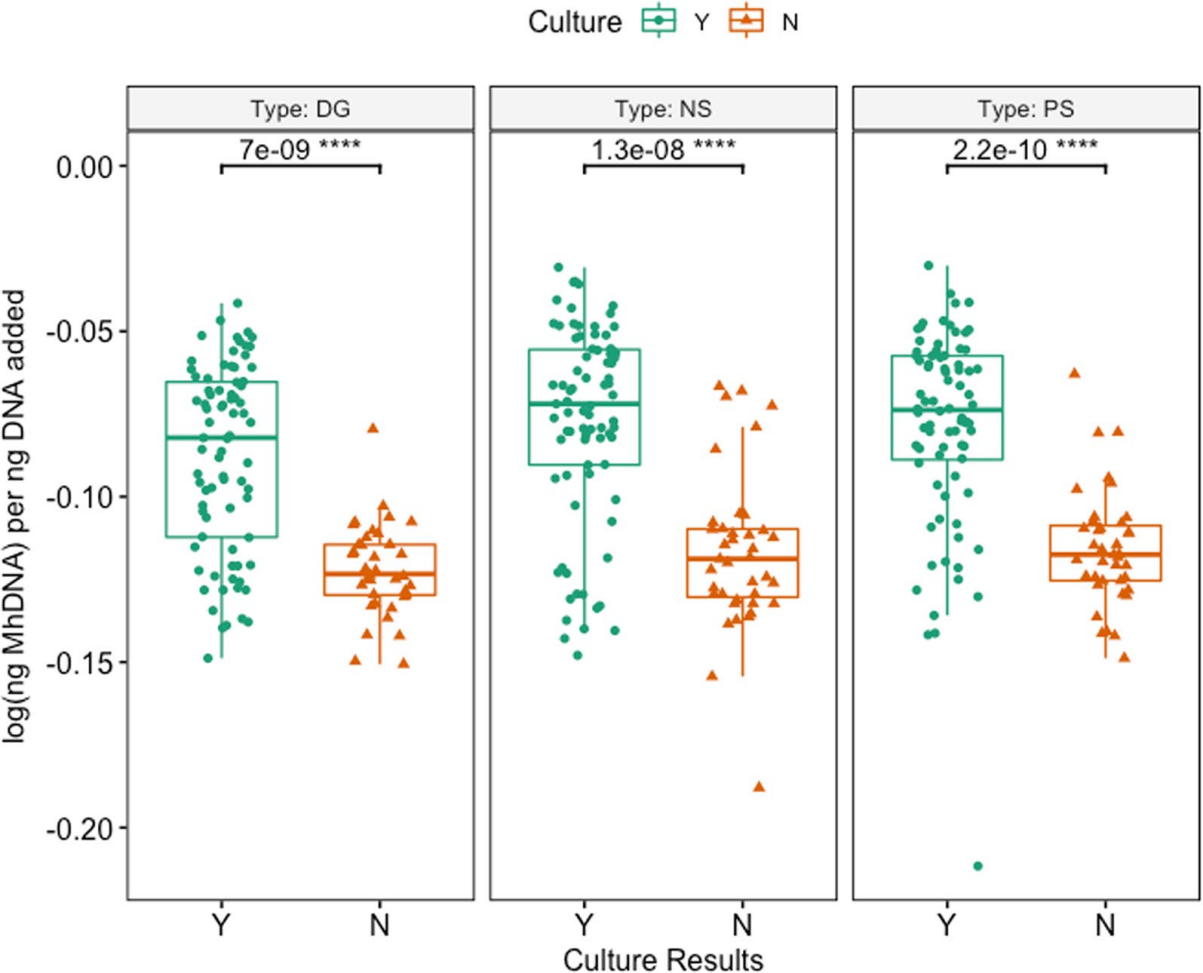
Box and whisker plots of log(ng *Mh* DNA) per ng DNA added vs sampling group, separated by Swab Type (Wilcoxon rank-sum test, *P* < 0.05)

*Mh* was detected by qPCR in 65.0% (78/120) of all cattle: 50.4% (59/117) of DG, 61.7% (74/120) of NS, and 58.0% (69/119) of PS (Additional file 4: Table S5). *Mh* was identified in significantly fewer samples collected with DG swabs as compared to either NS and PS in both group 1 and group 2, with group 2 having significantly fewer animals *Mh* positive than group 1 for each swab (Additional file 4: Table S4; McNemar’s Chi-square, *P* < 0.05). Similarly, log(*Mh* DNA) per ng DNA added was significantly different among swab types (Fig. 2; Kruskal–Wallis test, *P* = 0.048), and median log(*Mh* DNA) per ng DNA added for DG was significantly lower when compared to both NS and PS (Additional file 2 & Fig. 2; pairwise Dunn test with Benjamini–Hochberg correction, *P* = 0.05 and *P* = 0.05, respectively). There was complete concordance among qPCR between swabs for 81.9% of animals (95/116); two concordant positive and 1 discordant negative result was found in 12.1% of animals (14/116), and 1 discordant positive result with 2 concordant negative results was identified in the remaining 6.0% of animals (7/116) (Table 2). When evaluating the swabs’ ability to identify *Mh* by qPCR in animals treated or not treated for BRD, there was no difference among swab types in either group of animals (Fig. 3; Kruskal–Wallis, *P* > 0.05). When evaluating the effect of *Mh* culture on the ability to identify *Mh* by qPCR, swabs that came from animals who were culture positive had significantly higher log(ng *Mh* DNA) per ng of DNA added to reaction compared to animals that were culture negative, and this was true for all swab types (Figs. 4, 5; Wilcoxon rank-sum test, *P* < 0.05).

### 16S rRNA gene sequencing

#### Overall differences in microbial community composition

**Fig. 6:**
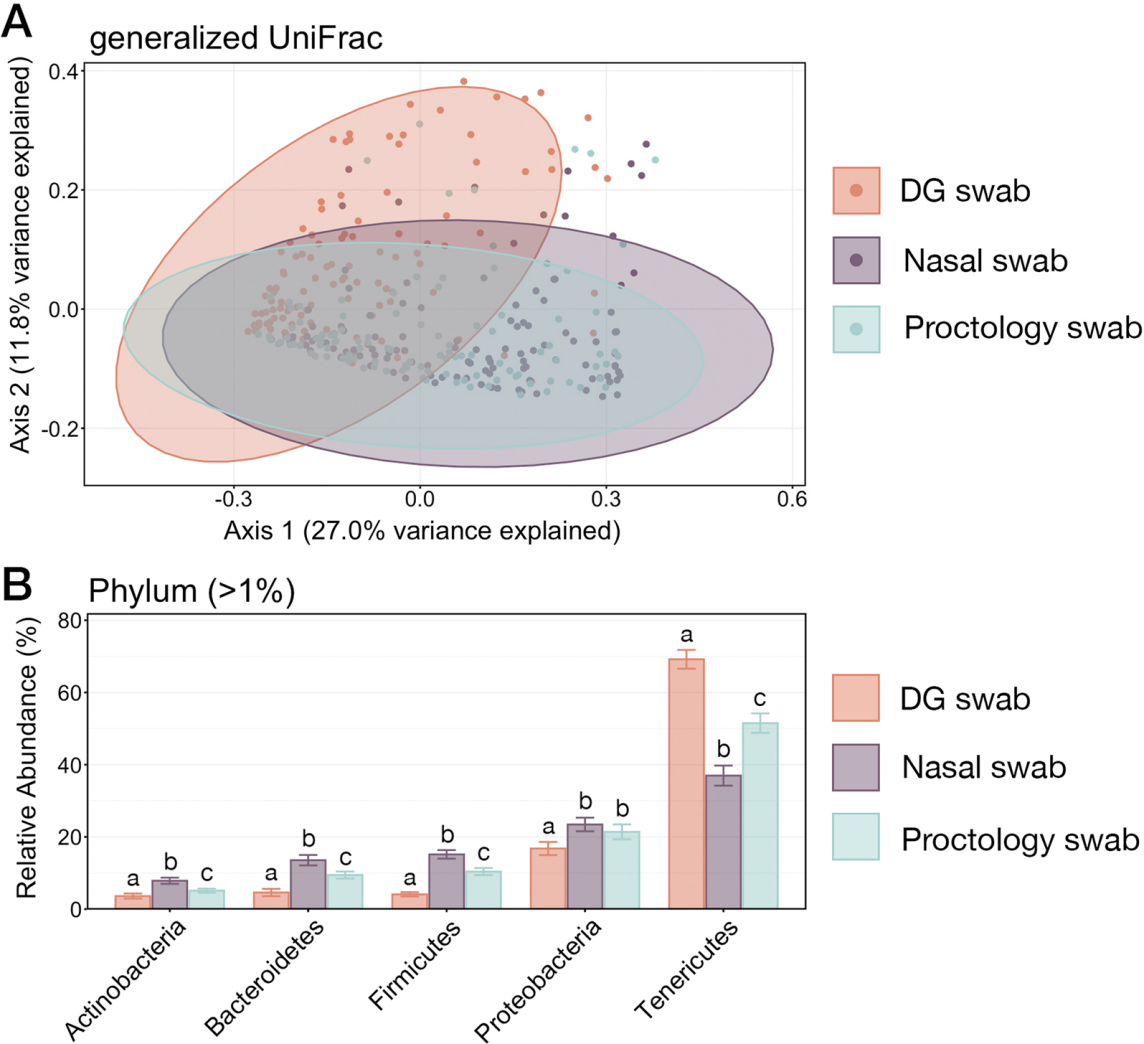
**A** Principal coordinates analysis (PcoA) of generalized UniFrac values illustrating differences in microbial community composition between samples collected with each swab type. The PCoA demonstrates clustering of ASVs from microbial communities collected with DG swabs, NS, or PS. Shaded areas represent 95% confidence ellipses for each swab type. Microbial community composition differed significantly between each community type (pairwise PERMANOVA with Benajmin-Hochberg correction, *P* < 0.005). **B** Barplot showing the relative abundances of the six phyla representing greater than 1% of the whole community illustrating the variation in microbial community structure across all samples. Error bars on the barplot demonstrate the standard error of the mean relative abundance for each of the six phyla when sampled using DG swabs, NS, or PS. Significant differences between relative abundances as collected with each swab type are illustrated by different letters (Pairwise Wilcoxon rank-sum test with Benjamini–Hochberg correction, *P* < 0.05)

The effect of sample collection method (i.e., DG, NS, or PS) on microbial community composition was analyzed with PERMANOVA and Principal Co-ordinates Analysis. Based on generalized UniFrac values, microbial community structured differed significantly between samples collected with each swab type (Additional file 4: Table S6; pairwise PERMANOVA with Benjamini–Hochberg correction; *P*-adj. < 0.05). However, PCoA illustrated that the samples collected with DG swabs had the most unique community structure, while the amount of variation explained by collecting samples with NS versus PS was exceedingly small (< 2%) and that those communities were very similar (Fig. 6).

To further compare differences in microbial communities resulting from the three sampling methods, the relative abundance of phyla representing more than 1% of the overall community across all samples were compared. Except for the relative abundance of Proteobacteria in samples collected using NS and PS, there were significant differences in the relative abundances of all six phyla among the different sample types (Fig. 6; pairwise Wilcoxon rank-sum test with Benjamini–Hochberg correction, *P* < 0.05). However, the prevalence of these six phyla followed the same order across all three swab types, with Tenericutes being the most abundant phyla followed by Proteobacteria, Firmicutes, Bacteroidetes, and Actinobacteria.

#### Characterizing microbial shifts related to clinical BRD

**Fig. 7:**
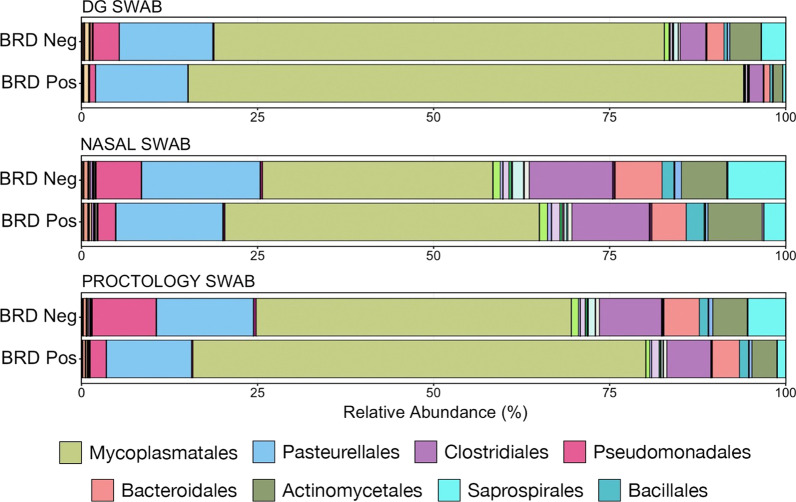
Bar plot illustrating the mean relative abundance of microbial orders within BRD negative or positive animals as sampled with DG swab, NS, or PS. Abundances were normalized to the total number of CSS-normalized ASVs within each sample. The 8 most abundant orders are displayed in the legend

**Fig. 8:**
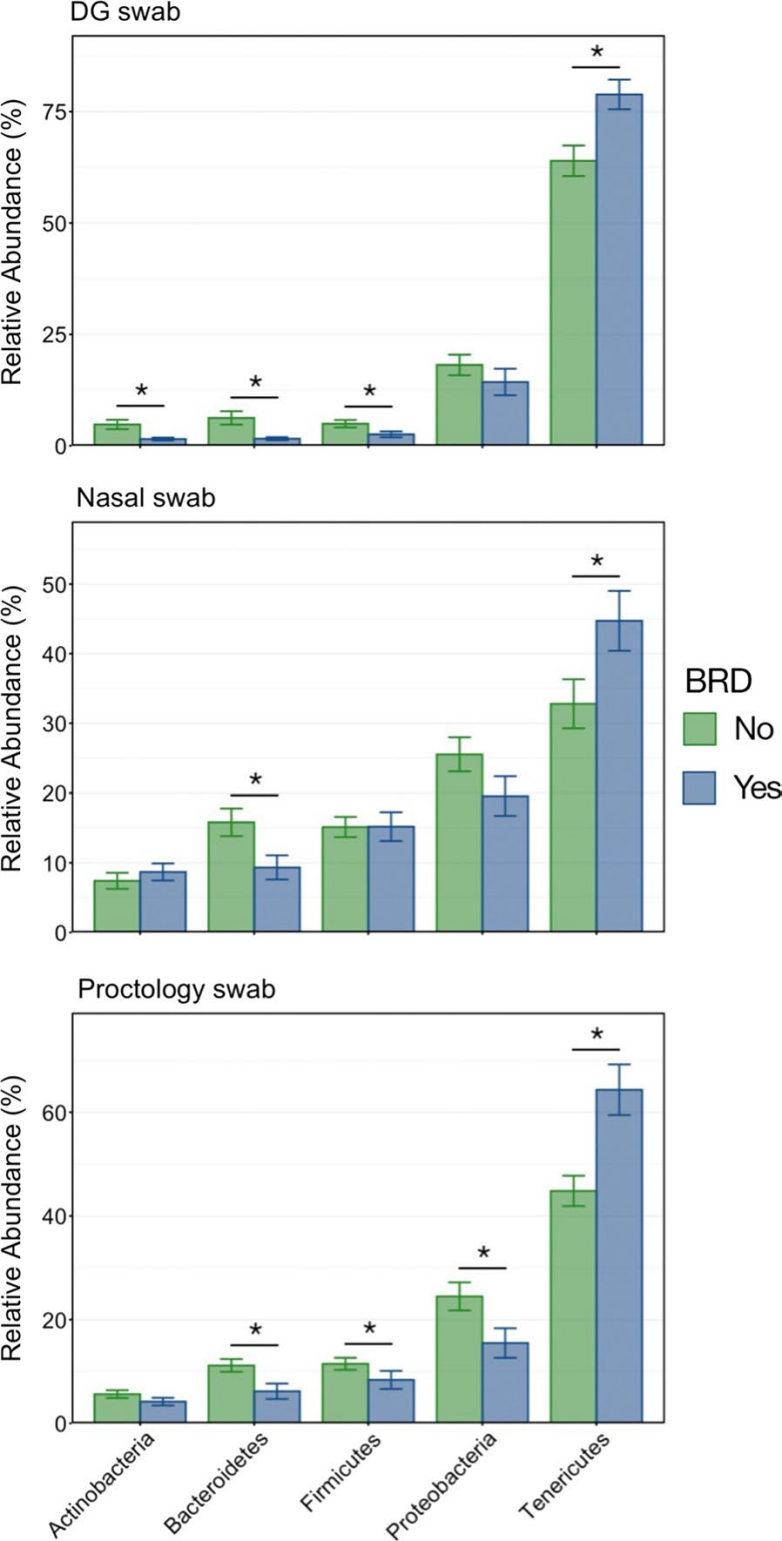
Bar plot demonstrating differences in the relative abundance of each of the 6 most abundant phyla in BRD negative and BRD positive animals as collected with DG swabs, nasal swabs, or proctology swabs. Error bars display the standard error of the mean. Significant differences among relative abundances within each phylum are noted with an asterisk (Kruskal–Wallis analysis of variance by ranks, *P* < 0.05)

Differences in microbial composition, as they related to the occurrence of BRD were visualized at the taxonomic level of order, based on the normalized proportion of ASVs within individual samples (Fig. 7). Each swab type demonstrated a similar shift between BRD-negative and BRD-positive animals: an increased relative abundance of the order Mycoplasmatales coupled with decreases in relative abundance of Pseudomonadales, Clostridiales, and Bacteroidales. Of the six phyla representing greater than 1% of the overall microbial community, four differed significantly in abundance between BRD-negative and -positive animals when sampled using DG swabs. Only two phyla differed if samples were collected using NS, while four differed significantly when sampled with PS (Fig. 8; Kruskal–Wallis analysis of variance on ranks, *P* < 0.05). All three collection methods demonstrated a significant difference in Tenericutes, which was the most abundant phylum across all samples and was almost exclusively composed of the order Mycoplasmatales. However, sample collection using NS was less effective in characterizing changes within less abundant phyla than samples collected using DG swabs or PS.

**Fig. 9:**
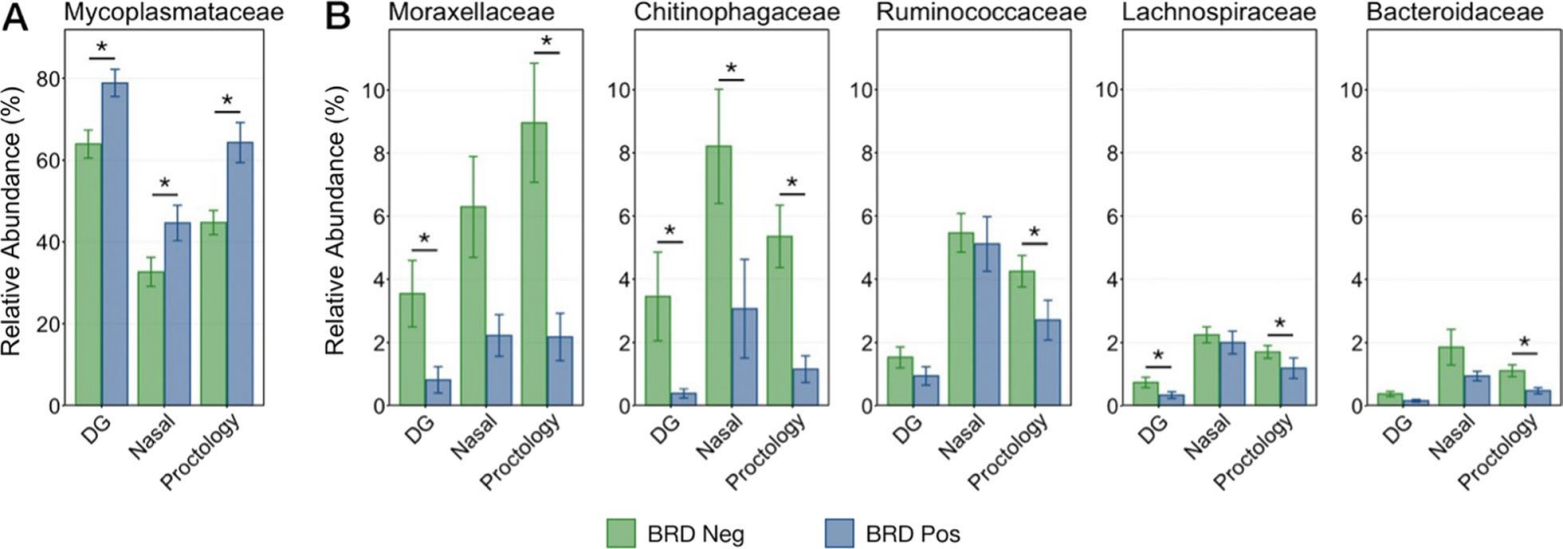
Bar plot demonstrating differences in the relative abundance of microbial taxa of interest within BRD negative and BRD positive animals as collected with DG swabs, nasal swabs, or proctology swabs. Error bars display the standard error of the mean. Significant differences among relative abundances within each phylum and swab type are noted with an asterisk (Kruskal–Wallis analysis of variance by ranks, *P* < 0.05). Note the difference in the relative abundance scale for 4A and 4B

To illustrate potentially important differences among the different sample types, the relative abundances were further examined for six families (Mycoplasmataceae, Moraxelleceae, Ruminococcaceae, Lachnospiraceae, Chitinophagaceae, and Bacteroidaceae) and three genera (*Mannheimia, Pasteurella*, and *Histophilus*) that were differentially abundant between BRD-negative and -positive animals, or were of specific clinical interest. Generally, the same trend within these taxa was observed across samples collected with all three swab types. The family Mycoplasmataceae was in significantly higher abundance in BRD-positive animals when sampled with all three swab types (Fig. 9A; Kruskal–Wallis analysis of variance on ranks; *P* < 0.05). Mycoplasmataceae was also overwhelmingly the most abundant ASV at the family level, representing over 50% of the total microbial population across all sample types and virtually 100% of all Tenericutes. Additionally, the relative abundance of the two genera of Mycoplasmataceae detected in this study were compared between BRD-positive and BRD-negative animals. *Mycoplasma* comprised the vast majority (> 97%) of Mycoplasmataceae, and its relative abundance was significantly higher in BRD positive animals in samples collected with any swab type (Additional file 3: Figure S6; Kruskal–Wallis analysis of variances on ranks; *P* < 0.05). The relative abundance of *Ureaplasma*, which was greatest in samples collected with DG swabs, did decrease in BRD positive animals (Additional file 3: Figure S6). However, because of its low abundance (~ 1%) and large variation among individual animals the difference was not significant within any swab type (Kruskal–Wallis analysis of variances on ranks; *P* > 0.05).

The families of Moraxellaceae (almost exclusively composed of the genus *Moraxella* and unclassified Moraxellaceae) and Chitinophagaceae demonstrated the largest decrease in relative abundance of taxa measured in BRD-positive animals (Fig. 9B). The decrease in Moraxellaceae was only significant when sampled using DG swabs and PS, while Chitinophagaceae decreased significantly using all three swab types (Fig. 9B; Kruskal–Wallis analysis of variance on ranks; *P* < 0.05). The families Ruminoccaceae, Bacteroidaceae, and Lachnospiraceae also decreased in BRD-positive animals, though the differences were smaller and largely only significant when samples were collected using PS (Fig. 9B; Kruskal–Wallis analysis of variance on ranks; *P* < 0.05). Due to their clinical relevance, the relative abundance of the genera *Mannheimia, Pasteurella, Histophilus* and their family Pasteurellaceae were also compared between BRD-negative and -positive animals, but there were no differences in abundances among any of the sampling methods (Additional file 3: Figure S7; Kruskal–Wallis analysis of variance on ranks; *P* > 0.05).

#### Characterizing microbial shifts in *M. haemolytica* culture-positive animals

**Fig. 10:**
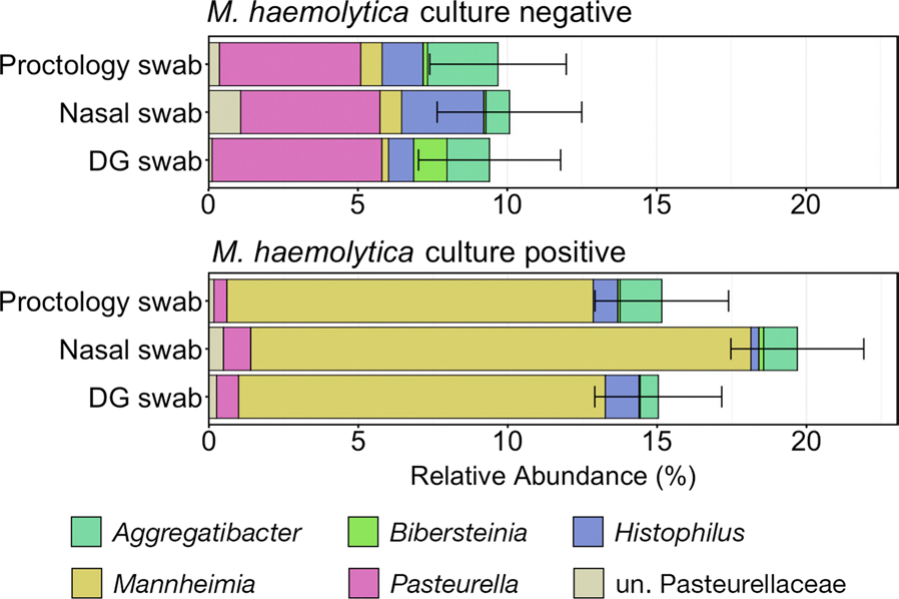
Bar plot showing the relative abundances among all classified taxa for five Pasteurellaceae genera and unassigned Pasteurellaceae ASVs within *M. haemolytica* culture-negative and culture-positive animals, as collected with DG swabs, nasal swabs, or proctology swabs. Error bars demonstrate the standard error of the mean relative abundance of Pasteurellaceae. The six most abundant genera across all samples are displayed in the legend. Abbreviations: un., unclassified

As *Mh* is widely considered one of the most important respiratory pathogens of cattle, the different sampling methods were compared regarding the ability to capture differences in microbial abundances between *Mh* culture-positive and culture-negative animals. ASVs associated with the genus *Mannheimia* represented an average of only 0.56% ± 0.19% (SEM) of the total microbial community in samples collected from *Mh* culture-negative animals, but significantly increased to an average abundance of 13.7% ± 1.22% (SEM) in culture-positive animals (Fig. 10; Kruskal–Wallis analysis of variance on ranks; *P* < 0.05). While *Mannheimia* increased, both *Pasteurella* and *Histophilus* decreased significantly in abundance within *Mh* culture-positive animals for all sample types (Fig. 10; Kruskal–Wallis analysis of variances on ranks; *P* < 0.05). However, the sample collection method (DG swab, NS, or PS) did not impact the abundance of Pasteurellaceae or *Mannheimia,* as there were no differences within animals of the same *Mh* culture status (Fig. 10; pairwise Wilcoxon rank-sum test with Benjamini–Hochberg correction, *P* > 0.05).

## Discussion

This unique study compared three sample collection methods (deep-guarded nasopharyngeal swabs, unguarded 16-inch proctology swabs, and unguarded 6-inch nasal swabs) to identify *Mh* and characterize changes in the microbial community structure within the context of bovine respiratory disease, using a combination of culture-dependent and culture-independent (16S rRNA gene sequencing and qPCR). The results were largely equivalent when comparing samples collected by DG, NS, or PS relative to the detection of *Mh* or characterization of the microbial community composition. This has important ramifications for researchers studying microbial communities of the upper respiratory tract of live cattle because of the significant logistical issues of sample collection under conditions of commercial cattle production. While there were differences among the sample types in statistical significance of the results, the trends in *Mh* detection and characterization of *Mh*/BRD-associated shifts in microbial communities were consistent regardless of sample collection method. As BRD is one of the leading causes of morbidity and mortality in cattle and one of the most common reasons for treatment with antimicrobial drugs [1], improving methods for investigating BRD pathogens within the context of entire microbial communities is critical to furthering our understanding of this disease, as well as efficiently conducting relevant surveillance. The results presented here provide researchers with justification for choosing a simpler sampling method to characterize bovine respiratory tract microbial communities and the pathogens playing important roles in BRD; however, it is important to note that the concordance was not perfect among sampling methods. Therefore, depending on the clinical or research questions of interest, researchers may elect to use different sampling strategies. Similar studies in different production classes or management systems are encouraged to determine if these simpler sampling methods are applicable in other contexts (stocker, dairy, or cow-calf operations; no metaphylaxis; etc.). Investigation of these swabs’ comparibility in isolation of other bacteria of interest in BRD, such as *Pasteurella multocida*, *Histophilus somni*, or *Mycoplasma bovis* should also be considered.

Variation in the structure of microbial communities inhabiting different segments of the respiratory tract of cattle (e.g., nasopharynx or bronchoalveolar) has been described previously [18]. DG swabs have been used by investigators to specifically sample the nasopharynx without contamination from the more rostral nasal passage, but they are more logistically challenging to use and are more expensive than other swabs used in this study. The short NS employed in this study were easier to use but only sampled the most rostral few inches of the nasal passage. The PS swabs sampled both the nasal passage and the nasopharynx, sampling a region of the upper respiratory tract that was effectively a combination of the regions sampled with the DG and NS. The DG samples exhibited the most unique microbial community structure (Fig. 6a), but these differences in community structures were largely attributable to differences in abundance of shared taxa and not the presence of different taxa, and the rank of most abundant taxa was the same among all swab types (Fig. 6b). Interestingly, the relative differences in the abundance of common microbial taxa when comparing NS to PS to DG samples suggests that an ecological gradient may exist within the bovine respiratory tract, as NS swab only rostral nasal cavity, DG sample the only nasopharynx, and PS collect a ‘composite’ sample. Ecological gradients (i.e., pH, salinity, temperature) are well-established drivers of microbial community structure in environmental microbiology [42–44], but this concept is largely unexplored within the context of respiratory tracts. Results from this study regarding culture and molecular-based detection of *Mh* are consistent with previous research demonstrating that recovery of the upper respiratory tract is consistent with the culture of the lower respiratory tract in acute cases of BRD and at the group level [11, 20, 21, 45]. However, given that this study only explored differences within upper respiratory tract samples, we cannot remark about the consistency in results among DG, PS, and NS and lower respiratory tract sampling methods.

Importantly, the trends for microbial taxa of interest in BRD-positive or BRD-negative animals were essentially the same for each swab type (Fig. 7). *Mycoplasma bovis* and *Mannheimia haemolytica* are the bacterial pathogens most commonly associated with BRD [9, 46–48] and in this study *Mycoplasma* had significantly higher relative abundance in BRD-positive animals, regardless of sample type, which is consistent with a previous report of recently weaned beef calves [49]. Interestingly, there was no difference in the relative abundance of *Mannheimia* or any other Pasteurellaceae genera believed to be important in BRD at this level of the respiratory tract, in contrast with patterns of pathogen detection in the lower respiratory tissues in cattle with BRD that die [8, 50]. One potential reason for these differences is that, in the present study, some cattle were sampled before treatment for disease and even before showing signs of disease in some cases; however, previous work has shown that microbial community is different at arrival in animals that go on to have BRD compared to those animals that remain healthy and that *Mannheimia* did not have increased relative abundance in diseased animals [19].

It is also important to note that tildipirosin metaphylaxis could have affected the nasopharyngeal microbiome. The nasopharyngeal microbiome of calves treated with tulathromycin, another macrolide, has been shown to recover by day 12 after administration [51]. There is little information on the duration that tildipirosin administration would affect the nasopharyngeal microbiota; however, it is reasonable to speculate that tildipirosin may have a longer duration of effect on the microbiome than tulathromycin, due to the longer half-life of tildipirosin in lung tissue of 10 days [52] compared to 8.75 days for tulathromycin (Draxxin product label, Zoetis). Holman et al. also demonstrated that there was a large effect on the nasopharyngeal microbiome within 2–5 days after administration [14], meaning BRD treatment shortly before 14 DOF could have some effect on the nasopharyngeal microbiome observed in this study compared to others, as well.

Differences relative to BRD status for other taxonomic orders were less expected as these taxa are not typically considered to be important members of respiratory flora (Fig. 9B). However, the decreased abundance of gut-associated taxa such as Ruminococcaceae, Chitinophagaceae, Bacteroidaceae, and unclassified Clostridiales may be the result of decreased rumination leading to decreased transfer to the upper respiratory tract in animals with BRD, which typically have decreased appetite [3].

The high prevalence of antimicrobial resistance in *Mh* isolates was consistent with previous studies involving upper respiratory culture of beef cattle at about 14 days after metaphylactic treatment with long-acting macrolide antibiotics [17, 53]. This high frequency of isolation of multidrug resistance is consistent with MALDI genotyping, as genotype 2 is more commonly associated the presence of antimicrobial resistance genes [54]. Clawson et al. also note that these resistance genes in genotype 2 are commonly associated with an integrative conjugative element (ICE). ICEs are mobile genetic elements (MGE) that can transfer to naïve cells via conjugative transfer, but also integrate into the genome of the bacterial host [55]. The presence of antimicrobial resistance genes on a MGE capable of inter-species and inter-genera transfer [56] may explain the similarity among sample types regarding isolation of MDR *Mh,* and highlights the importance of studying antimicrobial resistance in *Mh* and other BRD pathogens within the context of entire microbial communities and other BRD pathogens.

Culture and qPCR only targeted *Mh*, but the use of 16S rRNA sequencing was a very useful and synergistic investigation approach as it allowed both focused and broad-based investigation of the composition of the respiratory microbiome. However, it was still limited in the investigation of microbes affecting BRD occurrence as it did not allow investigation of viral agents that are believed to be highly important in the occurrence of this multifactorial disease. Incorporation of additional molecular diagnostics would allow an even broader metagenomic investigation of all microbes (bacterial, archeal, viral) of the respiratory tract, in addition to host factors affecting BRD occurrence [57–59].

## Conclusions

Results of this study showed that the three sampling methods evaluated provided highly comparable results regarding evaluation of *M. haemolytica* recovery by culture, detection by qPCR, and for characterization of microbial community structure using 16S rRNA gene sequencing. The results support the conclusion that, when samples are being collected for *M. haemolytica* culture or qPCR, NS or PS can be chosen over DG, to provide comparable results with less expense and greater ease of sampling*.* Further, relative differences in microbial community structure that were found identified in relation to BRD status were reflected similarly for all three sample types. In contrast, variations in abundance of some taxa (e.g., for the genus *Mycoplasma*) identified by different swab types suggests that DG swabs may be the most appropriate for studies characterizing these organisms, particularly if there is interest in comparing results to previous research using DG swabs. Future work should compare these sampling techniques in calves or cattle from different production sectors, as well as comparing the results of upper respiratory tract sampling with PS with lower respiratory tract sampling methods such as tracheal wash and/or bronchoalveolar lavage.

## Supplementary Information

**Additional file 1.** Standard Curve of qPCR.

**Additional file 2.** Culture and qPCR Results.

**Additional file 3.** Supplementary Figures.

**Additional file 4.** Supplementary Tables.

## Data Availability

All data generated or analyzed during this study are included in this published article and its additional information files.

## Abbreviations

ASV: Amplicon sequence variants
BRD: Bovine respiratory disease
DG: Deep-guarded swab
*Mh*: *Mannheimia haemolytica*
NS: Nasal swab
PS: Proctology swab
qPCR: Quantitative polymerase chain reaction
