# Supplementary material for: Does swab type matter? Comparing methods for Mannheimia haemolytica recovery and upper respiratory microbiome characterization in feedlot cattle
Source: Anim Microbiome. 2022 Aug 13;4:49. doi: 10.1186/s42523-022-00197-6 (PMC9375289; doi:10.1186/s42523-022-00197-6)
Supplement: Supplementary file 3 — Additional file 3. Supplementary Figures. [file 42523_2022_197_MOESM3_ESM.docx]

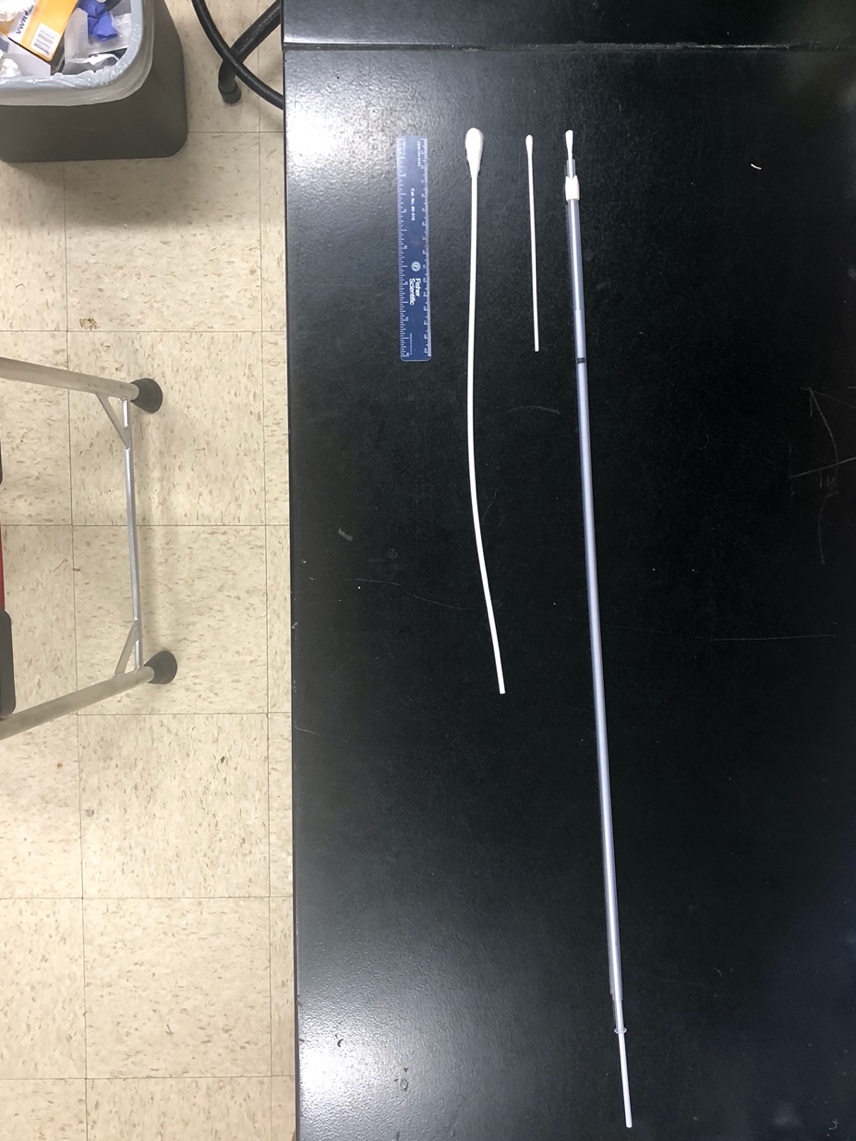


**Figure S1.** Photograph of swabs used for upper respiratory tract sampling. From top to bottom: double-guarded swab (DG), nasal swab (NS), and proctology swab (PS);15 cm ruler included for scale.


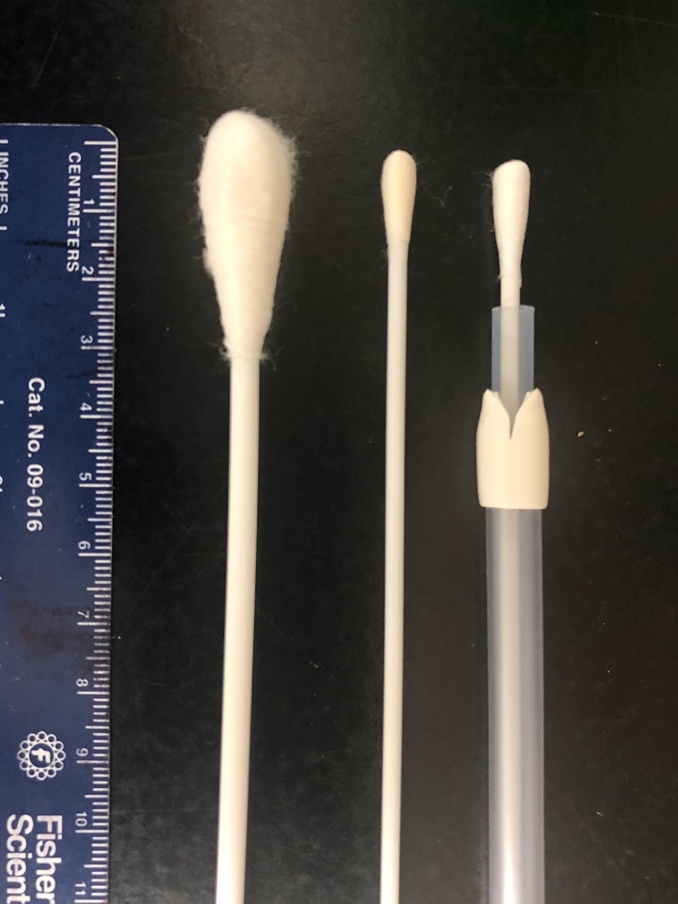


**Figure S2.** Photograph of swab tips used for upper respiratory tract sampling. From top to bottom: double-guarded swab (DG), nasal swab (NS), and proctology swab (PS); 15 cm ruler included for scale.


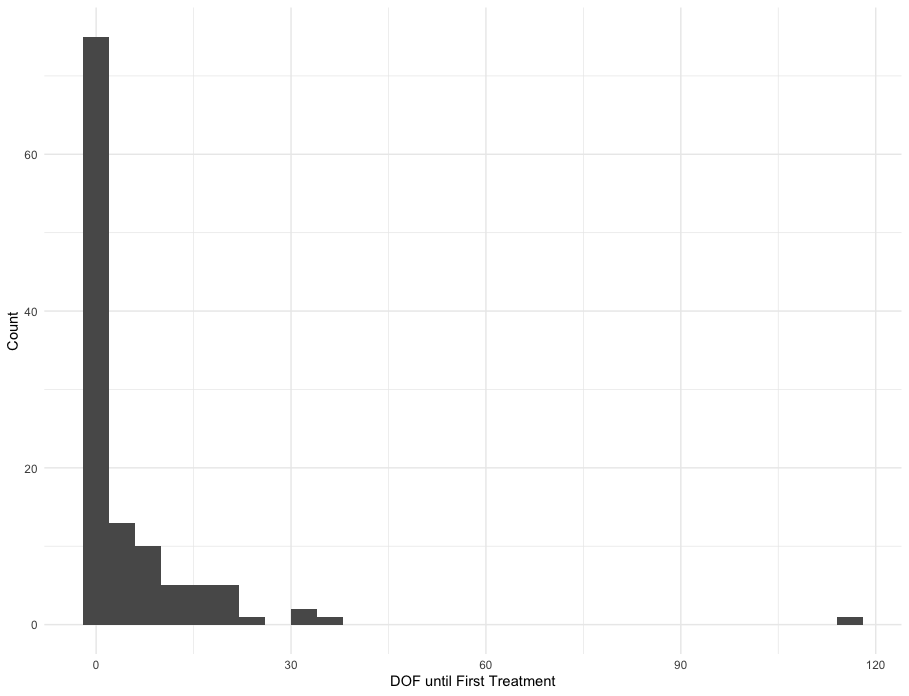


**Figure S3.** Histogram of days on feed at feedlot until first treatment for BRD for 120 cattle sampled at 14 DOF.


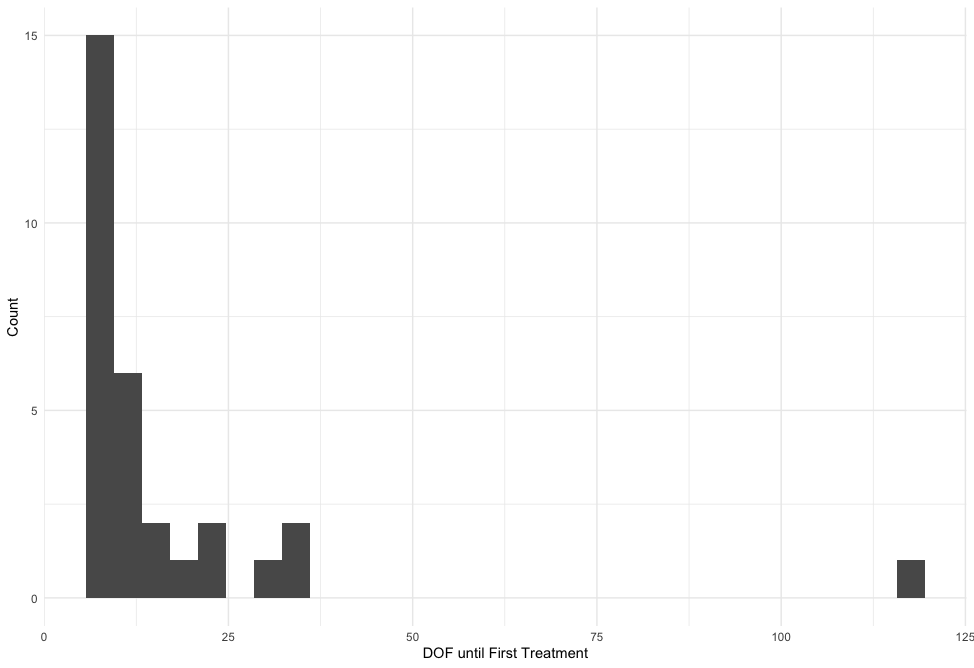


**Figure S4.** Histogram of days on feed at feedlot until first treatment for BRD for first group of 60 cattle sampled at 14 DOF.


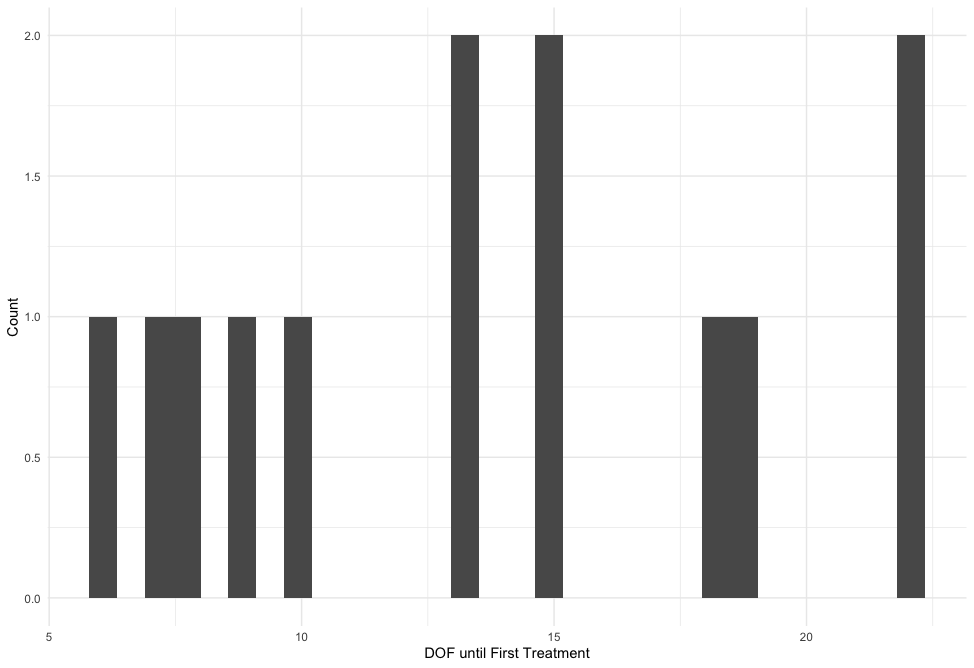


**Figure S5.** Histogram of days on feed at feedlot until first treatment for BRD for second group of 60 cattle sampled at 14 DOF.


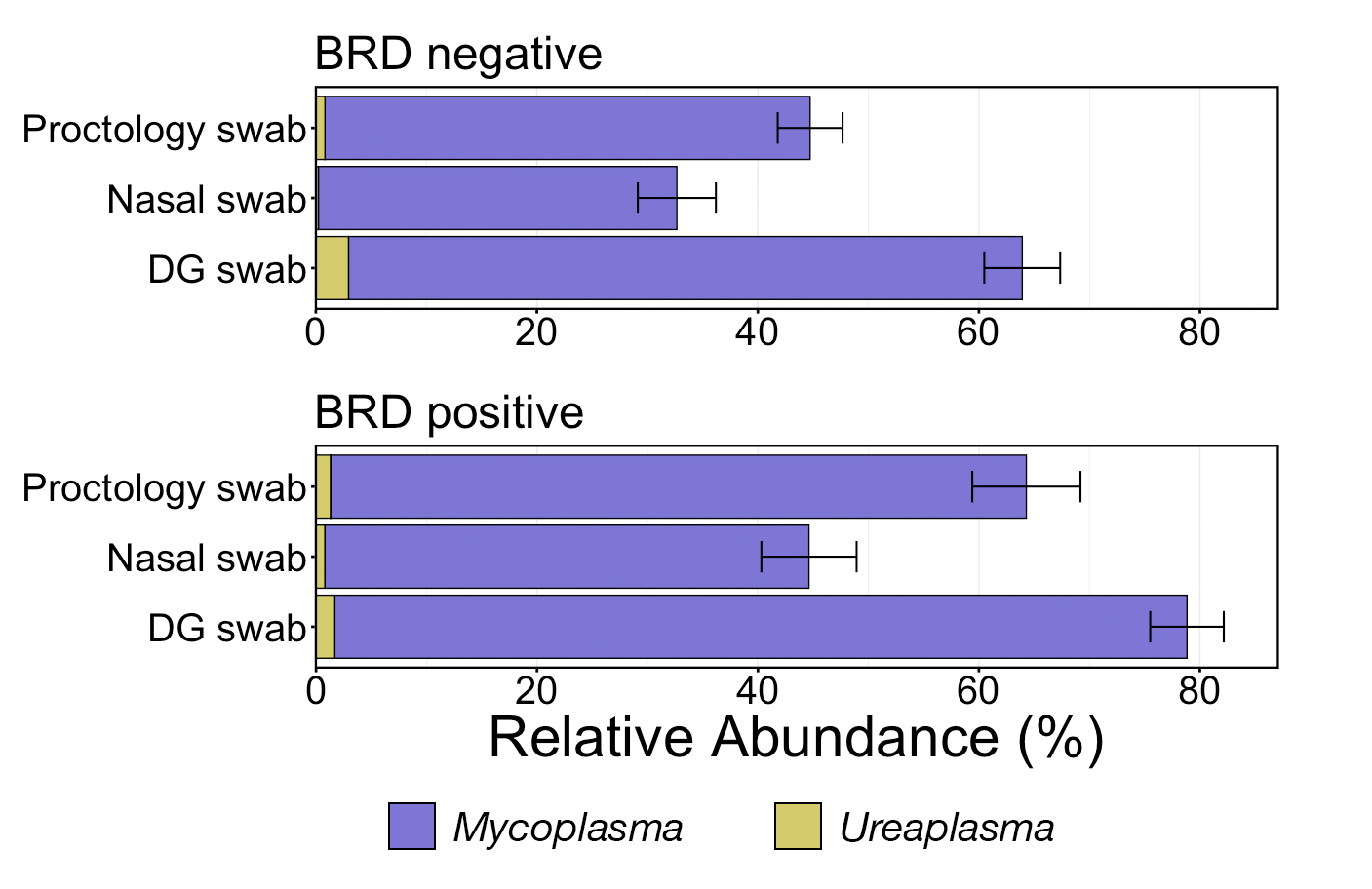


**Figure S6.** Bar plot demonstrating the relative abundances of the two detected Mycoplasmataceae genera ASVs within BRD negative and BRD positive animals as collected with DG swabs, nasal swabs, or proctology swabs. Error bars demonstrate the standard error of the mean relative abundance of Mycoplasmataceae. Taxa representing greater than 0.1% of the overall community across all community across all samples are displayed in the legend.


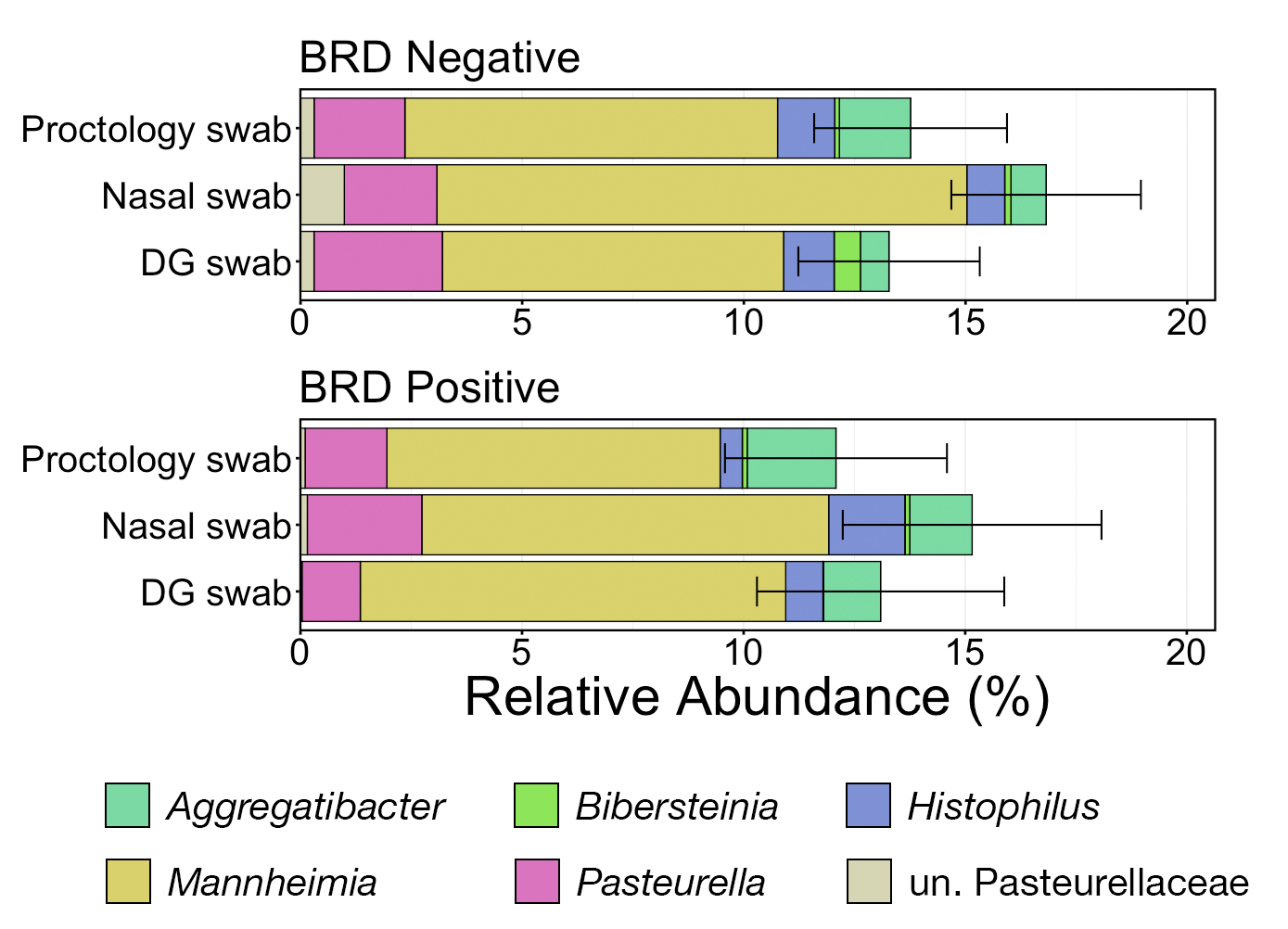
**Figure S7.** Bar plot demonstrating the relative abundances of the eight detected Pasteurellaceae genera and unclassified Pasteurellaceae ASVs within BRD negative and BRD positive animals as collected with DG swabs, nasal swabs, or proctology swabs. Error bars demonstrate the standard error of the mean relative abundance of Pasteurellaceae. The six most abundant genera across all samples are displayed in the legend. Abbreviations: un., unclassified
