# Supplementary material for: Does swab type matter? Comparing methods for Mannheimia haemolytica recovery and upper respiratory microbiome characterization in feedlot cattle
Source: Anim Microbiome. 2022 Aug 13;4:49. doi: 10.1186/s42523-022-00197-6 (PMC9375289; doi:10.1186/s42523-022-00197-6)
Supplement: Supplementary file 4 — Additional file 4. Supplementary Tables. [file 42523_2022_197_MOESM4_ESM.docx]

**Table S1.** Processing protocols, date, and product used at arrival to feedlot

| Study Day | Management | Product | Dose | Company |
| --- | --- | --- | --- | --- |
| -1 | Individual ID | N/A | N/A | N/A |
|  | Metaphylaxis | Tildipirosin (Zuprevo) | 4 mg/kg, SQ | Intervet, Inc. |
|  | Clostridial Vaccine | Calvary 9 | 2 mL | Merck Animal Health |
|  | Respiratory Vaccine | Once PMH | 2 mL | Merck Animal Health |
|  | BVD PI Testing | N/A | N/A | N/A |
|  | Growth Implant | Zeranol (Ralgro) | 36 mg | Merck Animal Health |
|  | Deworm | Ivermectin + corsulon (Ivermectin Plus)  albendazole (Valbazen) | 1 mL/110 lb, SQ  10 mg/kg, PO | Durvet, Inc.  Zoetis |
|  | Sex | N/A | N/A | N/A |
|  | Weight* | N/A | N/A | N/A |
| 0 | Weight* | N/A | N/A | N/A |
|  | Castration | N/A | N/A | N/A |
|  | Pain Control | Meloxicam | 1.1 mg/kg, PO | N/A |
|  | Other Study Measures | Rump fat, rib fat, rib eye ultrasound | N/A | N/A |
|  | Sorting | N/A | N/A | N/A |

*Weights recorded for study were mean of day -1 and day 0 weights.

**Table S2:** Clinical illness score (Perino & Apley, 1998, Vet Clinics of North America-Food Animal Practice). Animals with clinical scores $\geq$2 and rectal temperature $\geq$ 40 ^°^C were treated according to feedlot protocol*.

| Clinical Illness Score | **Description** | **Appearance** |
| --- | --- | --- |
| 0 | Normal | Normal |
| 1 | Slightly ill | Gaunt, nasal/ocular discharge |
| 2 | Moderately ill | Gaunt, nasal/ocular discharge, lags behind other animals in the group, cough, labored breathing |
| 3 | Severely ill | Purulent nasal/ocular discharge, labored breathing, not responsive to human approach |
| 4 | Moribund | Near death |

*First treatment: florfenicol (Nuflor, Merck Animal Health, Madison, NJ); second treatment:enrofloxacin (Baytril, Bayer Animal Health, Shawnee Mission, KS); third treatment: ceftiofur crystallin free acid (Excede, Zoetis, Kalamazoo, MI). All treatments given at label doses for therapy.

**Table S3.** Proportion of 16S rRNA gene sequence reads that were classified at each taxonomic rank.

|  | Taxonomic rank | | | | |
| --- | --- | --- | --- | --- | --- |
|  | Phylum | Class | Order | Family | Genus |
| Reads classified | 99.61% | 99.60% | 99.49% | 97.11% | 81.31% |

**Table S4.** Isolation rate of *M. haemolytica* from double guarded (DG), nasal (NS), and proctology (PS) swabs, separated by group (n = 60 per group). Values in the same column with different superscripts indicate significant difference (Chi-square, *P*<0.05)

| Group | DG | NS | PS | Total |
| --- | --- | --- | --- | --- |
| 1 | 40^a^ | 40^a^ | 42^a^ | 122 (68.1 %) |
| 2 | 27^b^ | 26^b^ | 26^b^ | 79 (43.9 %) |
| Total | 67 | 66 | 68 | 201 (56.0 %) |

**Table S5.** *M. haemolytica* identification rate by qPCR of each swab type, separated by group (n = 60 per group). Values in the same column with different superscripts indicate significant difference (Chi-square, *P*<0.05). Significant differences by swab type are bolded (McNemar Chi-square, *P<*0.05).

| Group | DG | NS | PS | Total |
| --- | --- | --- | --- | --- |
| 1 | **36^a^** | 46^c^ | 43^a^ | 125 (70.6 %) |
| 2 | **23^b^** | 28^b^ | 26^b^ | 77 (43.0 %) |
| Total | 59 | 74 | 69 | 200 (56.7 %) |

**Table S6**. PERMANOVA and PERMDISP results from comparisons between bovine upper respiratory tract microbial communities using generalized UniFrac distances and 9999 permutations. Significant results are bolded.

| **Test** | **Df** | **SS** | **Pseudo-F** | **R^2^** | **p-adj.** | **PERMDISP (p-adj.)** |
| --- | --- | --- | --- | --- | --- | --- |
| DG swab vs nasal | 1 | 2.770 | 24.534 | 0.098 | **0.0002** | 0.793 |
| DG swab vs proctology | 1 | 1.624 | 14.948 | 0.063 | **0.0002** | 0.120 |
| Nasal vs proctology | 1 | 0.378 | 3.664 | 0.016 | **0.0020** | 0.066 |

Abbreviations: Df, degrees of freedom; SS, sum of squares; p-adj., adjusted p-value
